# Is insomnia disorder associated with time in bed extension?

**DOI:** 10.5935/1984-0063.20200089

**Published:** 2020

**Authors:** Yoann Birling, Guixia Li, Mingxian Jia, Xiaoshu Zhu, Jerome Sarris, Alan Bensoussan, Jian Wang, Paul Fahey

**Affiliations:** 1 Western Sydney University, NICM Health Research Institute - Penrith - NSW - Australia.; 2 Guang’anmen Hospital of China Academy of Chinese Medical Sciences, Psychology and Sleep Department - Beijing - Beijing - China.; 3 Beijing University of Chinese Medicine, Chinese Medicine - Beijing - Beijing - China.; 4 Western Sydney University, School of Science and Health - Penrith - NSW - Australia.; 5 University of Melbourne, Professional Unit, The Melbourne Clinic, Department of Psychiatry - Melbourne - VIC - Australia.

**Keywords:** Sleep, Behavior, Sleep Initiation and Maintenance Disorders

## Abstract

**Objective:**

There is a lack of evidence for extension of time in bed behaviors (i.e., getting to bed earlier, going out of bed later, staying in bed while awake and napping) as perpetuating factors of insomnia. The aim of this study is to assess if insomnia disorder is associated with extension of time in bed behaviors.

**Methods:**

150 good sleepers and 173 insomniacs were recruited between December 2017 and June 2018. A cross-sectional survey was performed using the Wang Insomnia Integrated Questionnaire.

**Results:**

Bedtime, rising time and time in bed were not different between good sleepers and insomniacs (Cohen’s d, <0.01, 0.07, 0.07, respectively; all p>0.05) and were not correlated with insomnia severity (all p>0.05). Staying in bed while awake during the night and in the morning where both different between good sleepers and insomniacs (Cohen’s d, 1.33 and 0.85, respectively; all p<0.001) and were positively correlated with insomnia severity (all p<0.001). Napping was more frequent (p<0.01) among good sleepers (63.3%) than insomniacs (48.6%) and a predictor of good sleep (p<0.01).

**Conclusion:**

Going to bed earlier and getting out of bed later do not seem to be associated with insomnia. Staying in bed while awake during the night and in the morning are associated with insomnia but could be only signs of insomnia symptoms. Limiting time in bed to prevent insomnia might and suppressing insomniacs’ napping behavior to treat insomnia might not be effective.

## INTRODUCTION

Cognitive Behavioral Therapy for Insomnia (CBT-I), which was only a promising alternative to pharmaceutical treatment 30 years ago, is now widely recognized as the first-line treatment for insomnia disorder^1^. Two of the major reasons for being the first-line treatment is that cognitive and behavioral changes lead to more sustained improvements and provoke fewer side effects than biological treatments such as Benzodiazepine Receptor Agonists (BzRAs)^2^.

The theoretical base for CBT-I is that chronic insomnia is caused by perpetuating behavioral and cognitive factors^3^. CBT-I aims at changing these factor using cognitive and behavioral techniques^4^. Extension of time in bed was one of the first perpetuating factors to be proposed^5^. The extension of time in bed is considered to be a strategy used by insomniacs for coping with daytime fatigue and increase the opportunity for sleep, though conversely perpetuating the insomnia symptoms because of the mismatch between sleep opportunity and sleep ability^6^. The behaviors involved in time in bed extension (called below “extension behaviors”) include absolute extension behaviors (i.e., going to bed earlier, getting up later, and napping) and relative extension behaviors (i.e., staying in bed while awake during the night or in the morning).

The main evidence for extension behaviors being perpetuating factors of insomnia disorder is that Sleep Restriction Therapy (SRT), a therapy that targets extension behaviors by limiting insomniacs’ time in bed, is considered effective for the treatment of insomnia disorder^7^. However, SRT might improve sleep just by increasing the homeostatic drive and resetting the circadian cycle^6^, and not by suppressing extension behaviors. While being widely reported^6^, there is a lack of evidence that extension behaviors are indeed perpetuating factors of insomnia disorder^3^. This remains an important question in terms of behavioral prevention and treatment of insomnia.

In order to assess if insomnia is associated with extension behaviors, a questionnaire survey was conducted on insomniacs and good sleepers. This study focuses on the following questions: are extension behaviors more present in insomniacs compared to good sleepers? Are they more present during insomnia compared to before having insomnia? Are they associated with insomnia severity? Are they associated with insomnia duration? Are they more present in good sleepers with insomnia history compared with the ones without history?

## MATERIAL AND METHODS

### Design

This study is a cross-sectional survey using a questionnaire to collect data from two samples, i.e. the Insomnia Disorder (ID) group and the Good Sleepers (GS) group. The participants were surveyed between December 2017 and June 2018. The study was approved by the ethics committee of Guang’anmen Hospital (Beijing, China).

### Survey tool

**Figure 1:**
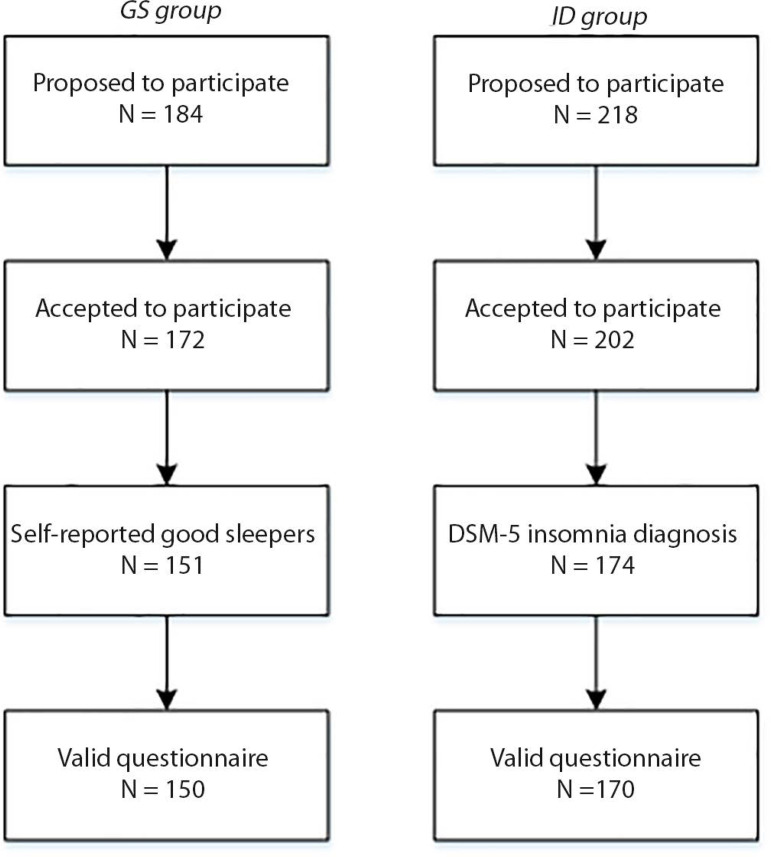
Participant flowchart.

This survey was conducted using the Wang Insomnia Integrated Questionnaire (WIIQ). The WIIQ was designed for the assessment of insomnia in the Chinese population^8^. It includes items covering demographics, sleep behavior assessment, insomnia history, cognitive assessment and questions specific to the patient’s education and psychological trauma^8^. The WIIQ has been validated for the assessment of insomnia severity, the insomnia severity score ranging from 0 to 23 with a high score indicating severe insomnia^9^. In addition to the demographics, the items used in this study are bedtime (BT), rising time (RT), time in bed awake at night (i.e., total time spent awake in the bed between the sleep onset and the final awakening; TIBN), time in bed awake in the morning (i.e., time between last awakening and rising; TIBM), napping status (i.e., napping or not), time in bed during napping (TN), and insomnia duration (time since diagnosis). The insomniacs reported BT, RT, TIBN and TIBM for both before having insomnia and for the week before completing the survey (Figure 1).

### Study population

Participants from the ID group were recruited at the sleep medicine department of Guang’anmen Hospital (Beijing, China). Patients with insomnia complaints were interviewed by a psychiatrist specialized in insomnia and diagnosed according to the insomnia disorder criteria of the Diagnostic and Statistical Manual, version 5 (DSM-5)^10^. Participants from the GS group were people attending health-related conferences in a community center affiliated to the hospital who reported having no trouble sleeping at the time of the survey. Forms were distributed to all the attendees at the beginning of the conference.

### Procedures

Explanations on the purpose of the study were provided to the participants and they all provided informed consent. In addition to the questionnaire, the participants of the GS group were asked to report if they have ever had insomnia. Questionnaires containing at least two missing relevant items were excluded.

### Sample size and statistical analysis

The achieved sample size of 150 or more per group provides more than 80% power to detect differences in group means as small as 0.32 standard deviation: a small to medium effect size according to Cohen’s rule of thumb^11^. Subgroup analyses comparing groups of sizes 81 and 89 and comparing groups of 53 and 93 had 80% power to detect differences between means of 0.43 and 0.49 standard deviations respectively. Bedtimes and rising times were transformed from 24h format to number of hours from 11 PM and 7 AM respectively for analyses and back-transformed to clock times for presentation. Total time in bed (TIB) was calculated as the duration between bedtime and rising time. Differences between the two samples in terms of demographics were analyzed with independent t-tests for numeric measures and Chi-square tests for categorical measures.

The difference between the extension behaviors of insomniacs before insomnia and with insomnia was assessed with paired t-tests. The associations between extension behaviors and insomnia severity were assessed with Pearson’s correlation coefficients. Multiple regression (linear and logistic) was used to investigate the differences in sleep patterns between groups corrected for demographic variables. *P*-values less than 0.05 were interpreted as evidence of a statistically significant difference between groups and *p*-values of less than 0.10 were considered as approaching clinical significance. The data was analyzed with SPSS 25.

## RESULTS

### Participants’ characteristics

The insomniacs surveyed were older on average, more likely to be female and less likely to have undertaken higher education or to be employed than the good sleepers. Only education levels differed statistically significantly between groups (Table 1). In the ID groups, no participant had used CBT-I previously and 89 participants were currently under hypnotic medication.

**Table 1:** Comparison of the demographics between the two samples.

Characteristics	GS (N=150)	ID (N=170)	*p*
Age, M (SD)	43.27 (15.99)	46.39 (12.87)	0.055
Gender, female (%)	92 (61.3)	119 (69.4)	0.129
Profession, N (%)			0.270
Employed	102 (68.0)	106 (62.4)	
Other	41 (27.3)	56 (32.9)	
Unreported*	7 (4.7)	8 (4.7)	
Education, N (%)			0.027
High school and less	38 (25.3)	63 (37.1)	
Higher education	110 (73.3)	106 (62.4)	
Unreported*	2 (1.3)	1 (0.6)	
Civil status, N (%)			0.749
Married	122 (81.3)	136 (80.0)	
Not married	27 (18.0)	33 (19.4)	
Unreported*	1 (0.7)	1 (0.6)	

GS = good sleepers, ID=people with insomnia disorder

* Excluded from Chi-square analysis

### Between-groups differences

The two groups were found to have similar BT, RT and TIB (Table 2). Unsurprisingly, insomniacs spent more time awake in the bed at night than the good sleepers, both during the night and after the final awakening (extra 1.48 hours TIBM and 0.82 hours TIBM for the ID group, both *p*<0.001). The proportion of nappers was higher (*p*=0.013) in the GS group (n=95, 63.3%) than in the ID group (n=82, 48.2%), with an average time in bed for naps non-significantly higher among the good sleepers (0.15 hours, *p*=0.147) (Table 2). The statistical significance of the differences was similar when subgroups of ID with or without hypnotic medication were isolated, except for RT, which was later (df=229, *t*=-2.208, *p*=0.028) among the ID under medication (06:51am±01:40) than the GS (06:40am±01:02).

**Table 2:** Comparison of the extension behaviors between the two samples. Adjusted for age, gender, profession, education and civil status. For all tests df = 305.

Extension behavior	GS (n=150)	ID (n=170)	*t*	*p*
BT, Mean (SD), hh:mm	10:37pm (00:49)	10:38pm (01:07)	-0.845	0.399
RT, Mean (SD), hh:mm	06:40am (01:02)	06:46am (01:24)	-1.405	0.161
TIB, Mean (SD), h	8.06 (1.04)	8.14 (1.17)	-0.794	0.428
TIBN, Mean (SD), h	0.52 (0.64)	1.98 (1.54)	-10.082	<0.001
TIBM, Mean (SD), h	0.44 (0.59)	1.27 (1.34)	-6.801	<0.001
TN, Mean (SD), h	0.72 (0.85)	0.57 (0.86)	1.454	0.147

BT=bedtime, RT=rising time, TIB=time in bed, TIBN=time in bed awake at night, TIBM=time in bed in the morning, TN=time in bed during napping.

### Differences between before and with insomnia

The insomniacs self-reported going to bed about 9 minutes later on average and rising about 5 minutes later on average compared to prior to the onset of their insomnia. The BT approached statistical significance (*p*=0.085) but not the RT difference (*p*=0.479) (Table 3). After having insomnia, they were also staying 1.24 hours longer in bed awake during the night on average (*p*<0.001) and 0.9 hours in the morning on average (*p*<0.001) (Table 3). The statistical significance of these differences was maintained when ID with or without medication were isolated, except for BT difference, which was approaching significance for ID with medication (*p*=0.069) but not for ID without medication (*p*=0.711).

**Table 3:** Comparison of the extension behaviors Before Insomnia (BI) and With Insomnia (WI) in the ID group. Adjusted for age, gender, profession, education and civil status.

Extension behavior	BI (n=170)	WI (n=170)	*t*	*p*
BT, Mean (SD), hh:mm	10:29pm (00:45)	10:38pm (01:07)	-1.731	0.085
RT, Mean (SD), hh:mm	06:41am (01:02)	06:46am (01:24)	-0.710	0.479
TIB, Mean (SD), h	8.21 (1.05)	8.14 (1.17)	0.691	0.491
TIBN, Mean (SD), h	0.76 (0.75)	1.98 (1.54)	-10.261	<0.001
TIBM, Mean (SD), h	0.37 (0.64)	1.27 (1.34)	-8.846	<0.001

BT = bedtime, RT = rising time, TIB = time in bed, TIBN = time in bed awake at night, TIBM=time in bed in the morning.

### Influence on insomnia severity

In the full sample, insomnia severity was found to be not correlated to BT (r=-0.062, *p*=0.271), RT (r =-0.002, *p*=0.966), TIB (r=0.052, *p*=0.349), and TN (r=-0.058, *p*=0.299), but positively correlated to TIBN (r=0.603, *p*<0.001) and TIBM (r=0.442, *p*<0.001). In the ID group, in addition to the correlation with TIBN (r=0.342, *p*<0.001) and TIBM (r=0.295, *p*<0.001) insomnia severity was also correlated negatively with BT (r =-0.242, *p*=0.001) and RT (r=-.161, *p*=0.036), TIB (r=0.039, *p*=0.613) and TN (r=0.112, *p*=0.146) staying uncorrelated. The statistical significance of the correlations was maintained when ID with or without medication were isolated, except for the correlation between RT and insomnia (r=-0.054, *p*=0.629) for the no-medication subgroup. Insomnia severity was higher on average (df=305, *t*=2.407, *p*=0.017) among non-nappers (mean±sd, 9.42±7.26) than among nappers (7.17±7.46), the difference being not significant anymore (*p*=0.212 for GS and *p*=0.497 for ID) when the groups were analyzed separately.

### Influence of insomnia duration and history

In the ID group and in both the with-medication and no-medication subgroups, insomnia duration was not a predictor of extension behaviors (all *p*>0.05). In the GS group, 53 participants had a history of insomnia and 93 no insomnia history. Except for TIB, which was found to be about 16 minutes shorter on average (*p*=0.039) among the insomnia history group (7.89±1.05h) than among the no-history group (8.16±1.04h), having insomnia history was found to not be a predictor of the other extension behaviors (all *p*>0.05).

## DISCUSSION

In this study, we found no statistically significant evidence that insomniacs exhibit absolute extension behaviors, compared to good sleepers and before having insomnia. Napping is actually more frequent among good sleepers than among insomniacs. However, insomniacs do spend statistically and clinically significantly more time awake in bed compared to good sleepers and before having insomnia. These behaviors may simply reflect insomnia symptoms (i.e., frequent and long awakenings during the night and early morning awakenings). In addition to being positively correlated to the time spent awake in bed, insomnia severity was also negatively correlated with bedtime and rising time, i.e. bad sleep is associated with going to bed and getting up early. The extension behaviors were not associated with insomnia duration and were not more frequent in good sleepers with insomnia history. A subgroup analysis showed that insomniacs tend to go to bed and get up later, this tendency being associated with bad sleep.

These findings are consistent with a recent meta-analysis showing that TIB measured with polysomnography is not statistically different between good sleepers and insomniacs^12^. However, a study using the Consensus Sleep Diary (CSD) found a statistically longer TIB for insomniacs^13^, the difference found (16 min) having a relatively low clinical significance. This divergence between these two studies might be explained by a larger sample size and a lower variability in the CSD study. A longitudinal study using sleep diaries suggested that TIB is not extended in acute insomniacs^14^. BT, RT and napping were not reported in these studies.

In this study, we found no evidence that absolute extension behaviors (i.e., getting to bed earlier, getting out of bed later and napping) were associated with insomnia. One explanation could be that insomniacs do not adopt extension behaviors, contrarily to Spielman’s hypothesis^5,15^. It may also be because of disparities between insomnia patient subgroups. Worry about sleep, perceived lack of control and hopelessness about the ability to sleep are some of the main characteristics of insomnia patients^16-18^.

It is possible that some insomnia patients go to bed later and avoid napping as avoidance behaviors (i.e., avoiding the bed) and get up earlier when waking up and give up napping as resignation behaviors, masking the fact that extending their time in bed maintains insomnia for another subgroup. Though napping is usually considered to disturb night-time sleep by decreasing homeostatic drive^19-21^, napping and longer napping were statistically significantly associated with good sleep in this study. This inconsistency may be mediated by the timing of the nap^22^.

The behavioral changes (i.e., later bedtime and rising time) observed in the subgroup of insomniacs on hypnotic medication may be explained by the residual effect of hypnotic drugs^23^. Because of the residual effect of some hypnotic drugs during the early morning, these insomniacs may wake up later and therefore get up later. The homeostatic drive being lower in the evening, they may feel sleepy later and therefore go to bed later. The associations between these changes and bad sleep observed in this study could be explained by a lower homeostatic drive and a delay of the circadian rhythm.

These findings have several implications for insomnia prevention and treatment. Firstly, it supports the instruction to get up when waking up during the night and in the morning. Doing so the patient does not only avoid bad sleep conditioning, it also enhances the homeostatic drive and give to the insomniacs a sense of control on insomnia^19,24^. Secondly, it does not support getting to bed later and getting out of bed earlier as a prevention strategy for insomnia. SRT might still be used because of its action on sleep homeostasis and circadian cycle^6^. Thirdly, napping may not be a perpetuating factor of insomnia that needs to be discontinued to improve sleep. Indeed, napping has been shown to have several positive influences on daytime alertness, task performance, and mood^25-27^.

In order to determine the effect of staying awake in bed and napping on insomnia, prospective studies comparing acute insomnia patients who stay in bed or who do not while awake and who nap or who do not. To analyze the behavior changes happening during the early stages of insomnia, a sample of people at risk for insomnia could be followed during a few months using sleep diaries and/or actigraphy. A CBT-I trial with two groups, one with napping and the other without napping, would allow us to better understand the role of napping and potentially improve CBT-I efficacy. Finally, the changes in BT, RT and TIB following hypnotic medication should be measured and reported in future clinical trials on hypnotic drugs for insomnia.

This study is to the knowledge of the authors the largest survey studying several sleep behaviors in insomniacs and good sleepers. The collection of behaviors before having insomnia allows a direct comparison without the influence of covariates (e.g., age, gender). The main limitation of this study is to use a cross-sectional design, preventing to observe the behaviors changes through time. Between the two groups, differences which are coherent with the demographic characteristics of insomnia patients^28,29^ were observed. These differences were managed by including the demographics into the analysis models.

Getting to bed earlier and getting up later do not seem to be perpetuating factors of insomnia disorder, while staying awake in bed is associated with insomnia. In contradiction with the current theory, napping is associated with good sleep. Prospective and longitudinal studies are required to clarify these findings.
